# Supplementary material for: Key drivers of cloud response to surface-active organics
Source: Nat Commun. 2019 Nov 18;10:5214. doi: 10.1038/s41467-019-12982-0 (PMC6861266; doi:10.1038/s41467-019-12982-0)
Supplement: Supplementary file 1 — Supplementary Information [file 41467_2019_12982_MOESM1_ESM.pdf]

Key drivers of cloud response to surface-active organics:

Supplementary information

Lowe et al. (2019)

Supplementary note 1:

Supplementary Figure 3 shows surfaces similar to those presented in Fig. 3 of the main text with the updraft axis replaced by broad Aitken mode concentrations variations associated with natural variabilities reported in the literature[58,31] and assuming $w = 0.32$ m s^{-1}. Peak cloud droplet number concentration (CDNC) enhancements Δ_{CDNC} are found at the same organic mass fractions F_{org} as in Fig. 3: $F_{\mathrm{org}} = 0.6$ and 0.7 for marine average (MA) and boreal (HYY) cases respectively; the nascent ultrafine mode event[16] (NUM-event, NE) is omitted on account of its well-defined organic mass fraction and aerosol concentrations[16] (Table 1, Fig. 1a-b). In support of arguments made in the main text, sensitivities are raised throughout the parameter planes for cleaner accumulation modes, across all environments, by facilitating droplet activation of smaller, and therefore a greater fraction of the, Aitken/NU mode particles.

Similarly, Supplementary Fig. 4 shows the dependencies of Δ_{CDNC} on the approximate compressed film model parameters, the assumed pure organic surface tension γ_{org} and minimum organic film thickness δ_{min}. Across the three cases – MA, HYY and NE – the same qualitative response in Δ_{CDNC} is evident; for thinner minimum film thicknesses and lower pure organic surface tension components Δ_{CDNC} is increased since critical supersaturation s_c are further suppressed by lower γ_{org} values and to larger sizes by δ_{min}, facilitating activation of smaller particles. While the qualitative behavior is common across the cases, there are significant differences in the magnitude of Δ_{CDNC} elicited by the organic mass fraction F_{org} and aerosol size distribution shapes. Specifically, HYY and NE cases are expected to show larger Δ_{CDNC} responses, as compared with MA, on account of larger F_{org} prescriptions, while the NE case results in an order of magnitude larger Δ_{CDNC} due to its unique size distribution shape (Fig. 1, Table 1). The mechanistic explanation of large Δ_{CDNC} promoted by low accumulation mode and high Aitken/NU mode concentrations is detailed in the main text.

Supplementary note 2:

The aerosol number concentration size distribution data recorded during summertime (May-August) 2012 at Mace Head used in the main text spans particle radii 11 – 250 nm and is shown in Supplementary Fig. 5a. Application of an algorithmic fitting procedure[67], and assumption of a bi-modal distribution throughout, yielded the log-normal size distributions shown in Supplementary Fig. 5b and fitted log-normal aerosol size distribution parameters shown in Supplementary Fig. 6. While measurement-to-fit errors are relatively minor, the total measured concentrations in some instances are significantly overestimated by the sum of fitted modal concentrations. These discrepancies arise as a result of Aitken mode fits that have significant concentrations concentrated at sizes below the lower limit of the measured size range (11 nm). Supplementary Figure 7a shows how, as one might expect, this arises when the fitted Aitken mode geometric mean radius R_1 takes smaller values. The adjusted concentrations, determined by integration of the mode over the measured size range, typically deviates from the raw total fitted concentration when $R_1 < 15$ nm. The same issue does not appear in the accumulation mode (Supplementary Fig. 7b) owing to the measured size range extending well-beyond all fitted values of R_2. This adjustment procedure is crucial for the appropriate representation of size distributions in the (N_1, N_2)-plane (Fig. 4a in the main text for example). The resulting adjusted placement in the (N_1, N_2)-plane is shown as a frequency of occurrence histogram in Supplementary Fig. 7c.

Supplementary note 3:

The relevance of an updraft-based sensitivity analysis for real-world conditions is best served by utilising a distribution of updrafts to derive updraft-weighted CDNCs[68]. This avoids over-emphasised CDNC sensitivities accruing from high-valued, but infrequently occurring

instances of w. In that light, we here employ a Gaussian-type updraft probability density function $P(w, \sigma_w)$ (Supplementary note 6, Supplementary Equation 5) so as to realise a physically-justified diagnosis of a global parametric sensitivity ranking (GPSR) of firstly CDNC at 30 m above maximum supersaturation (as modelled by bulk Köhler (BK) and approximate compressed film (CF) models, separately), and secondly, the response Δ_{CDNC} at the same altitude. These GPSRs we denote: $SR_{BK,\sigma}$, $SR_{CF,\sigma}$ and $SR_{\Delta,\sigma}$ and are also performed using a single, fixed updraft velocity framework: $SR_{BK,w}$, $SR_{CF,w}$ and $SR_{\Delta,w}$ for the sake of comparison. In terms of input parameters to be ranked, these two sets differ only in that the fixed updraft w is exchanged for σ_w since w becomes a dummy variable to be integrated over (Supplementary Equations 4 and 5) when applying $P(w, \sigma_w)$.

In order to carry out the GPSRs we use the Sobol algorithm[25,26,27] that provides a method of diagnosing model parametric sensitivities in the form of a ranking conditioned on specified parameter uncertainty ranges through a variance decomposition of model output. Through repeated model evaluations for simultaneous perturbations to all parameters of interest Θ_j model sensitivity can be diagnosed including parameter interactions as well as individual, or local, parameter sensitivity assessments. From such a decomposition, sensitivity indices can be calculated iteratively as one explores the complete parameter space, here performed by Latin hypercube sampling. From the Sobol algorithm, first order Sobol indices that provide a measure of relative sensitivity, local to a given parameter i, may be determined,

$$S_i = \frac{\text{Var}(\text{E}[\varphi|\Theta_i])}{\text{Var}(\varphi)} \quad (1).$$

where φ is the model output of interest, and $\text{E}[\varphi|\Theta_i]$ the expectation value of φ conditioned on parameter i. Since these indices provide a relative contribution of individual parametric sensitivities, $1 - \sum_i S_i$ gives some indication of the degree of interaction effects present in

model evaluations of φ. Interaction indices of increasing order ($S_i, S_{ij}, S_{ijk}\ldots$) may similarly be calculated at increasing computational cost - in the case of D model parameters, iterative calculation of 2^D-1 indices would be required for to capture all parameter interactions. In order to maintain computational tractability, the global sensitivity analysis can be performed using the total Sobol indices[69], which include parameter interactions of all orders, calculated as,

$$S_{T,i} = \frac{\mathrm{Var}(\varphi) - \mathrm{Var}(\mathrm{E}[\varphi|\Theta_{\forall j\neq i}])}{\mathrm{Var}(\varphi)} \quad (2)$$

where φ is the model output of interest and $\mathrm{E}\left[\varphi\middle|\Theta_{\forall j\neq i}\right]$ is the expectation value of φ conditioned on variations in all parameters excluding Θ_i. Since these indices provide a relative contribution of global parametric sensitivities, $\sum_i S_{T,i} - 1$ gives an indication of the degree of non-additivity present in model evaluations of φ, $\sum_i S_{T,i} = 1$ indicating a completely additive model of φ. It should be cautioned however that interactions between any given pair of parameters (m, n) are accounted for in both $S_{T,m}$ and $S_{T,n}$ and thereby a double-counting of sensitivity is introduced into $\sum_i S_{T,i}$, a problem that can be alleviated by explicitly calculating indices of intermediate order for models of a suitably low number of parameters considered.

The parameter set Θ_j in the context of this study is taken to contain the bi-modal log-normal aerosol number concentration size distribution parameters, modal concentrations $N_{(1,2)}$, geometric radii $R_{(1,2)}$ and standard deviations $\sigma_{g,(1,2)}$; organic mass fraction $F_{org,(1,2)}$; compressed film model parameters the minimum film thickness δ_{min} and pure organic surface tension γ_{org}; and updraft velocity w or standard deviation σ_w when implementing $P(w, \sigma_w)$. Ranges for sampling of each parameter are taken from references in the main text that were used for the uncertainty analysis (Fig. 5) and given in Supplementary Table 1.

Supplementary Figs. 8 and 9 contain the six GPSRs for marine and boreal setups, respectively. For the single updraft cases (Supplementary Figs 8 and 9, a b), they show that the

majority of model variance in CDNC at 30 m above maximum supersaturation is dominated by uncertainty in the updraft velocity w, followed by aerosol number concentration size distribution parameters, particularly modal concentrations, for both environments irrespective of whether the BK or CF model was applied. This result corroborates findings previously reported[34] found similarly using a Monte Carlo Markov Chain (MCMC) based approach to global sensitivity analysis. When using the updraft PDF $P(w, \sigma_w)$ setup (Supplementary Figs 8 and 9, d e), the accumulation mode concentration N_2 dominates the sensitivity ranking, for both marine and boreal environments, followed by the updraft PDF width σ_w. Increased importance of the Aitken mode, particularly the concentration N_1 for the boreal case (Supplementary Fig. 8b), in the CF framework is indicated by the larger total index and is as expected as the CF model predicts lower smallest activated size r^* facilitating a greater Aitken mode sourced CDNC and enhancing parameter interactions.

$SR_{\Delta,w}$ and $SR_{\Delta,\sigma,}$ i.e. setups where $\varphi = \Delta_{\mathrm{CDNC}} = \left(\frac{\mathrm{CDNC_{CF}}}{\mathrm{CDNC_{BK}}} - 1\right) \times 100\%$, are given in Supplementary Figs 8 and 9, c and f. By performing the analysis upon Δ_{CDNC} total model variance is reduced as changes in $\mathrm{CDNC_{BK}}$ and $\mathrm{CDNC_{CF}}$ due to perturbations in size distribution parameters, for example, will largely be negated in terms of CDNC predictions using BK and CF models. In contrast, the organic mass fraction F_{org} becomes significantly more important in this setup since an increase (decrease) in organic mass serves to simultaneously decrease (increase) $\mathrm{CDNC_{BK}}$ due to the more hygroscopic inorganic component and increase (decrease) $\mathrm{CDNC_{CF}}$ due to the compressed film mechanism for reasonable organic mass fractions (see Fig. 3 and Supplementary Fig. 3). Similarly, compressed film parameters, the minimum film thickness and pure organic surface tension component, are inactive in determining $\mathrm{CDNC_{BK}}$ and influence $\mathrm{CDNC_{CF}}$ in the direction of perturbation (see also Supplementary Fig. 4). Interaction effects are likely to be compounded by the opposite direction of influence of F_{org} on $\mathrm{CDNC_{BK}}$ and $\mathrm{CDNC_{CF}}$, particularly interactions with parameters associated with the organic

fraction. These heightened parameter interaction effects are most strongly seen in the boreal cases (Supplementary Fig. 9c and f), in which $\sum_i S_{T,i} = 3.53$ and 2.18 for fixed updraft and PDF setups respectively, likely owing to the larger F_{org} values probed. Nevertheless, these are effects are also visible to a lesser degree in marine cases, $\sum_i S_{T,i} = 1.41$ and 1.05 for fixed updraft and PDF setups respectively.

Supplementary note 4:

Criteria on organic mass fraction F_{org}, updraft w and aerosol Aitken N_1 and accumulation N_2 mode concentrations for CDNC and albedo enhancements, $\Delta_{CDNC} > 10$ % and $\Delta_{\alpha} > 5$ %, by the surface phase that were established in the main text are summarised in Tables 3 and 4. Supplementary Figure 10 shows the fitted second order power laws (solid lines) detailed in the main text and derived from the original contours in Fig. 4 (Supplementary Fig. 10, dashed), which provide inequality relations on N_1 and N_2 (in cm^{-3}) for the respective indicators of significant cloud microphysical responses.

Supplementary note 5:

Supplementary Figure 11 shows probability distribution histograms of various microphysical and optical property predications made by the cloud model initialised with 5,000 Latin hypercube sampled[38] parameter combinations within ranges given in Table 2. A fixed cloud depth of 200 m and average cloud fractions ε_c = 0.7 and 0.5 for marine and boreal (land proxy) cases, respectively, were assumed in all simulations and the uncertainty analysis presented in Fig. 5 of the main text. The 5th – 95th percentile ranges and means (used in

calculation of Fig. 5) of these probability distributions are shown in Fig S12. The shortwave cloud radiative effect (SW-CRE) differential is determined by[66]

$$\Delta(F) = -\varepsilon_s \varepsilon_c \frac{F_0 \Delta(\alpha)}{4} \quad (3)$$

where $\Delta(\alpha)$ is the absolute albedo differential induced by the surface phase, as modelled by the approximate compressed film model relative to the bulk Köhler model, and ε_s is the Earth's surface coverage of ocean 0.7 (marine case) or land 0.3, (boreal, land proxy case). Estimates of the SW-CRE differential given in Figs. 5, and Supplementary Figs. 11 and 12 are calculated using Supplementary Equation 3 and all rely on the above assumed fixed average cloud fractions and depth. In reality, spatiotemporal variations in the cloud field will be influenced by mesoscale and meteorological phenomena not readily captured by the adiabatic cloud parcel model framework. Supplementary Figure 13 gives a simplistic illustration of how the SW-CRE and its uncertainty, as captured by the parcel model framework and simplifying assumptions, depends on the fixed average cloud fraction.

Supplementary note 6:

General circulation models (GCM) generally rely on application of updraft probability density functions $P(w, \sigma_w)$ (where w and σ_w are the updraft velocity and standard deviation in ms^{-1}) to capture the significant sub-grid-scale variability in real-world updraft velocities[68]. Droplet activation parameterisations within GCMs are called over a range of updraft velocities specified by $P(w, \sigma_w)$ to derive weighted cloud droplet number concentrations[68],

$$\overline{\mathrm{CDNC}} = \frac{\int_0^\infty dw.\mathrm{CDNC}(w).P(w,\sigma_w)}{\int_0^\infty dw.P(w,\sigma_w)}. \quad (4)$$

For the purpose of droplet activation, the functional form of $P(w, \sigma_w)$ is routinely taken to be the positive tail of a zero-mean Gaussian distribution of width σ_w for (strato)cumulus cloud types[71,72,73]

$$P(w > 0, \sigma_w) = \frac{1}{\sqrt{2\pi\sigma_w}} e^{-\frac{w^2}{2\sigma_w^2}} \qquad (5)$$

and typically, $\sigma_w =$ 0.2 - 0.6 ms^{-1} in measured updraft velocity distributions and GCM simulations, examples are shown in Fig S4a. Successful aerosol-cloud droplet closure has been performed on (strato)cumulus cloud types by using average updraft velocities w as effective characteristic values for parameterisation input[71]. For $\sigma_w =$ 0.4 ms^{-1} and a positive-tail Gaussian distribution, this approach yields[73], $\bar{w} = \sqrt{\frac{2}{\pi}}\sigma_w = 0.32$ ms^{-1}, which we have used throughout the main text, unless otherwise stated.

Supplementary Figures:

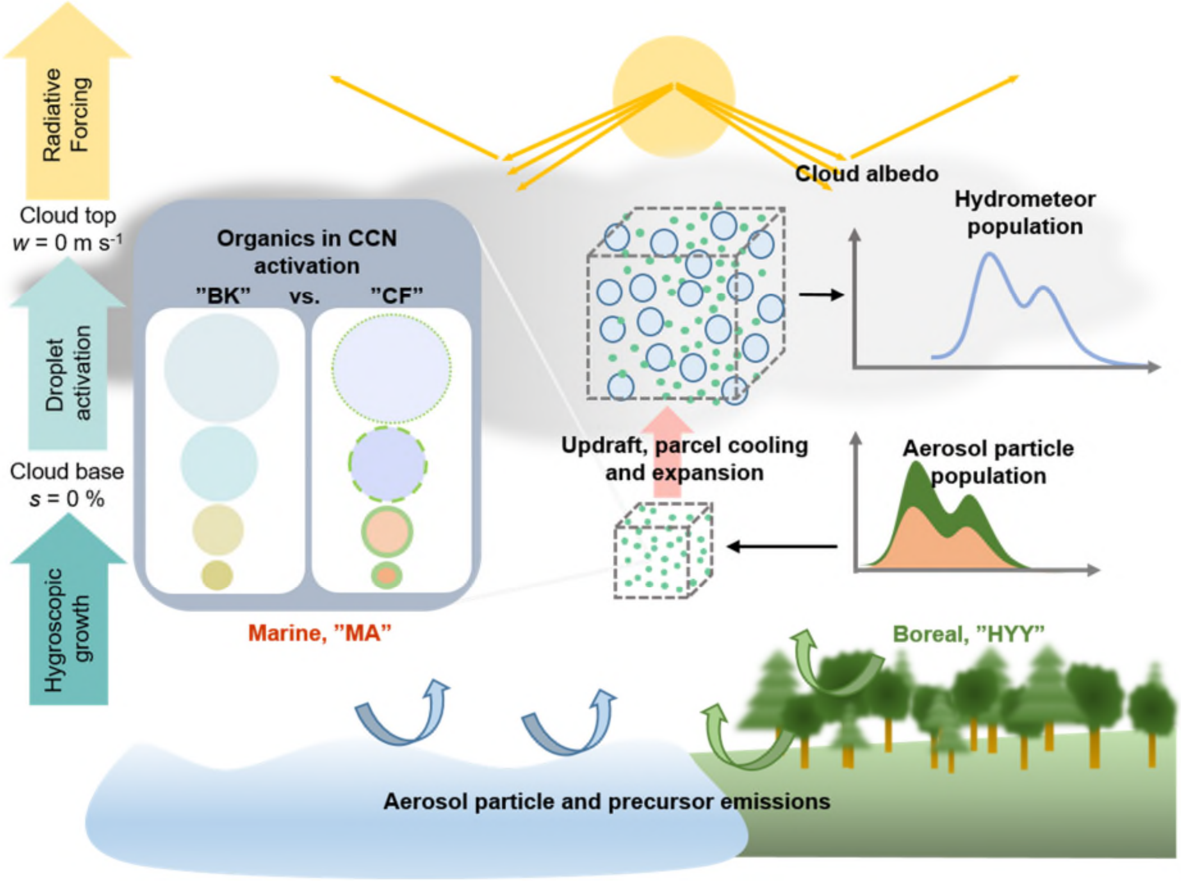

Supplementary Figure 1. Schematic representation of the phenomena studied. Using a cloud parcel model outlined in methods driven by aerosol size distribution data from two representative environments (MA, HYY) and realistic updraft schemes, we have investigated the importance of surface active organics (represented using the compressed film model, CF) on cloud microphysics (hydrometeor populations and cloud droplet number concentrations) and ultimately cloud albedo / radiative effects, as compared with the standard approach of using the traditional Köhler theory (denoted as bulk Köhler, BK).

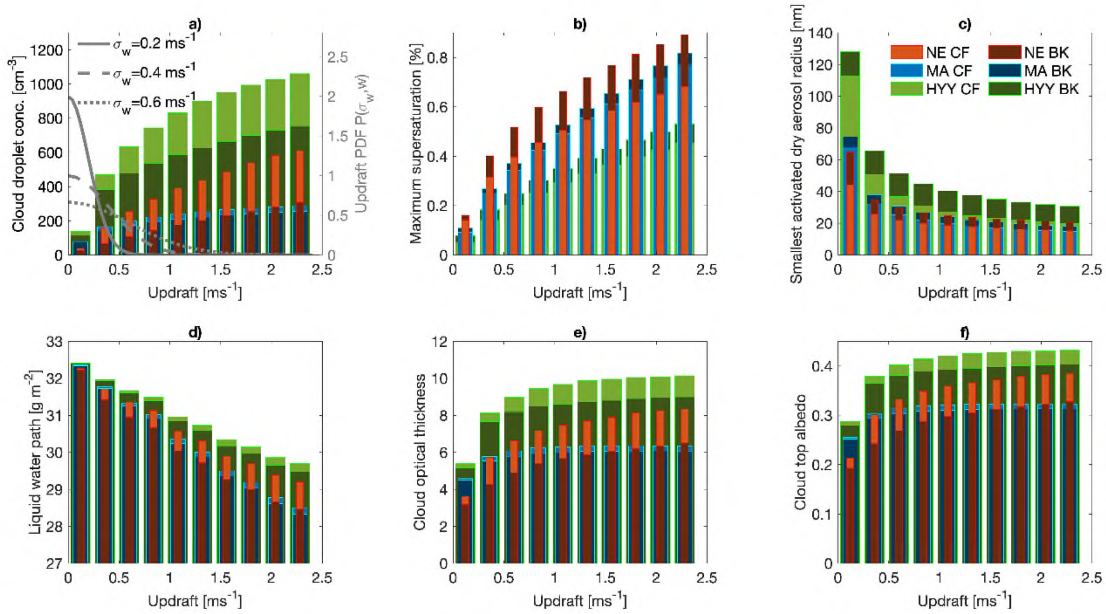

Supplementary Figure 2. Updraft probability distributions (a) and sensitivity of microphysical and optical properties indicated on y-axes (b-f). a) Typical positive-tail Gaussian-type updraft probability density functions $P(w, \sigma_w)$ (grey) in relation to modelled CDNC values as simulated for characteristic cases in the main text using the approximate compressed film (CF) and bulk Köhler (BK) model representations in the cloud parcel model.

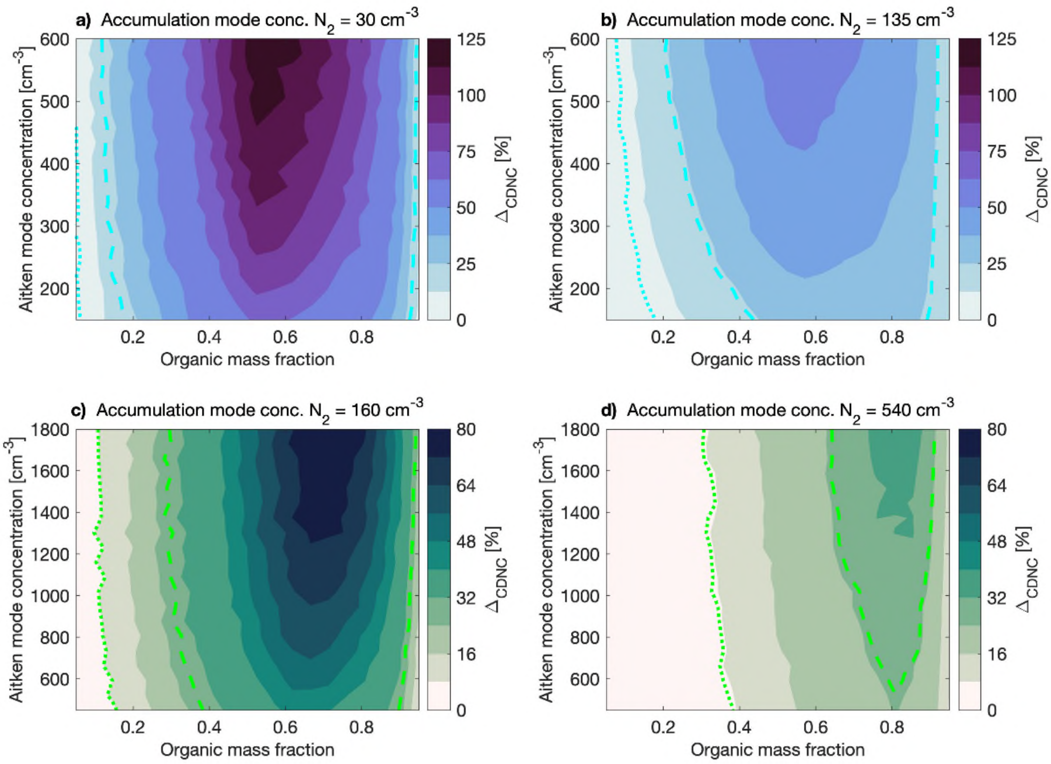

Supplementary Figure 3. Complementary surfaces to those shown in Fig. 3 of the main text, illustrating simultaneous cloud droplet number concentration enhancement to the surface phase Δ_{CDNC} dependencies on the organic mass fraction and Aitken mode concentration at 200 m above cloud base. All other input parameters are fixed to those of the characteristic simulations in Table 1 for marine MA (**a-b**) and boreal continental HYY (**c-d**).

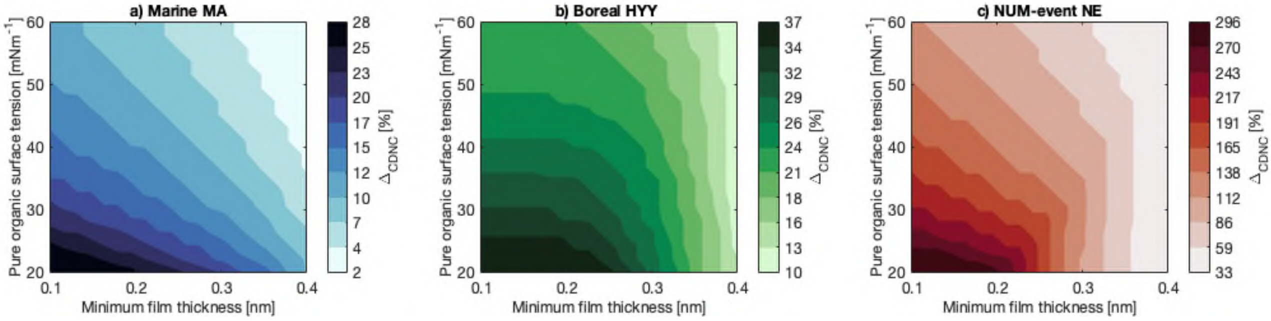

Supplementary Figure 4. Cloud droplet number concentration enhancement $\Delta_{\mathbf{CDNC}}$ surfaces illustrating dependencies on the compressed film parameters for MA (a), HYY (b) and NE (c) cases: pure organic surface tension component and minimum organic film thickness.

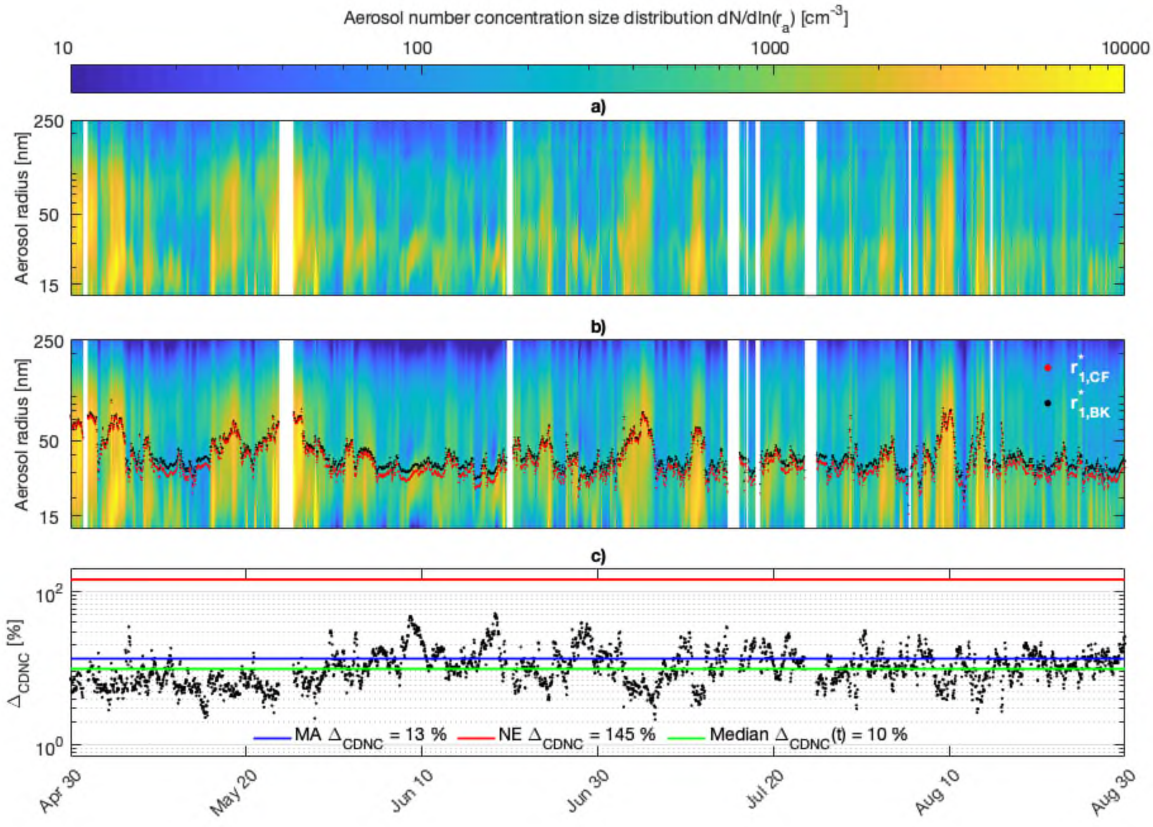

Supplementary Figure 5. Summertime coastal aerosol size distribution times series at Mace Head, 2012. a) Hourly averaged measurements of size distribution data made in the size range 10.5-250 nm using SMPS during May-August 2012. **b)** Size distributions fit to those given in a). Parcel model simulated smallest activated radii for bulk Köhler (black) and compressed film (red) models are overlain. **c)** Corresponding simulated CDNC response time series $\Delta_{\mathrm{CDNC}}(t)$ to the surface phase as represented by the compressed film model versus bulk Köhler model, assuming composition of MA simulation in Table 1 of the main text and an updraft value of 0.32 ms^{-1}.

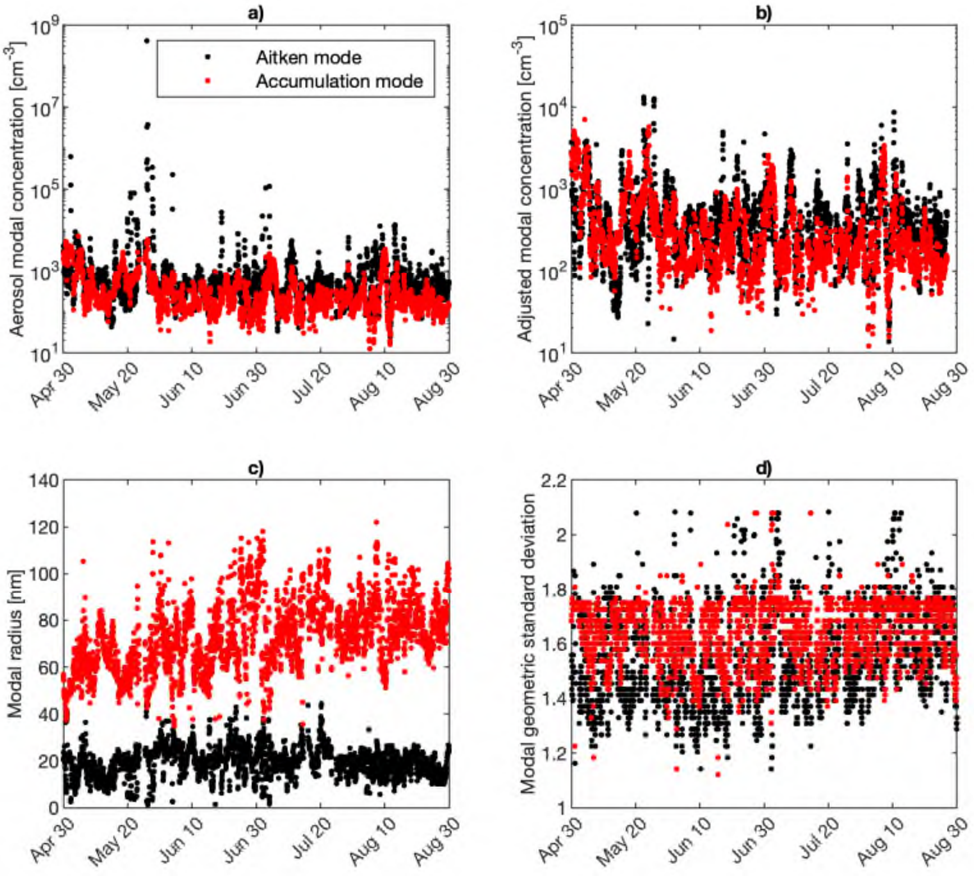

Supplementary Figure 6. Fitted aerosol size distribution parameters to coastal aerosol recorded at Mace Head during summer 2012. a) Fitted aerosol mode concentrations and their adjusted values in **b)** (see also Supplementary Fig. S9) corresponding to only particles contained within the measured size range during the time series shown in Supplementary Fig. 7. **c**) Fitted geometric mean radii. **d**) Fitted geometric standard deviations. Fitting performed using the algorithm developed by *Hussein et al.*[73].

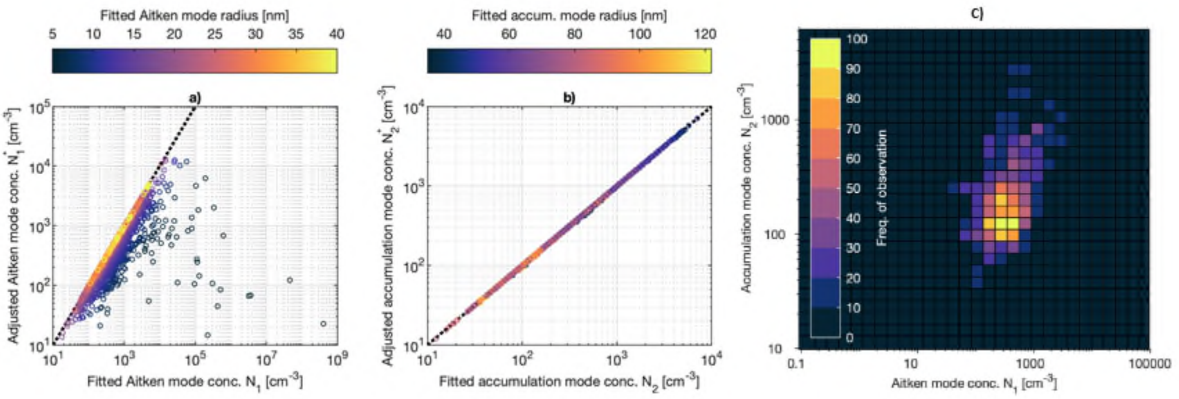

Supplementary Figure 7. Adjusted modal concentration fits correcting for shouldered modes. a) Adjusted Aitken mode concentration determined by integrating the fitted mode exclusively over the measured size distribution range 11-251 nm and a function of modal radius. **b)** As in a) but for the accumulation mode. **c)** Frequency of occurrence of fitted modal concentrations in the 2012 summertime Mace Head aerosol size distribution time series (Supplementary Fig. 5).

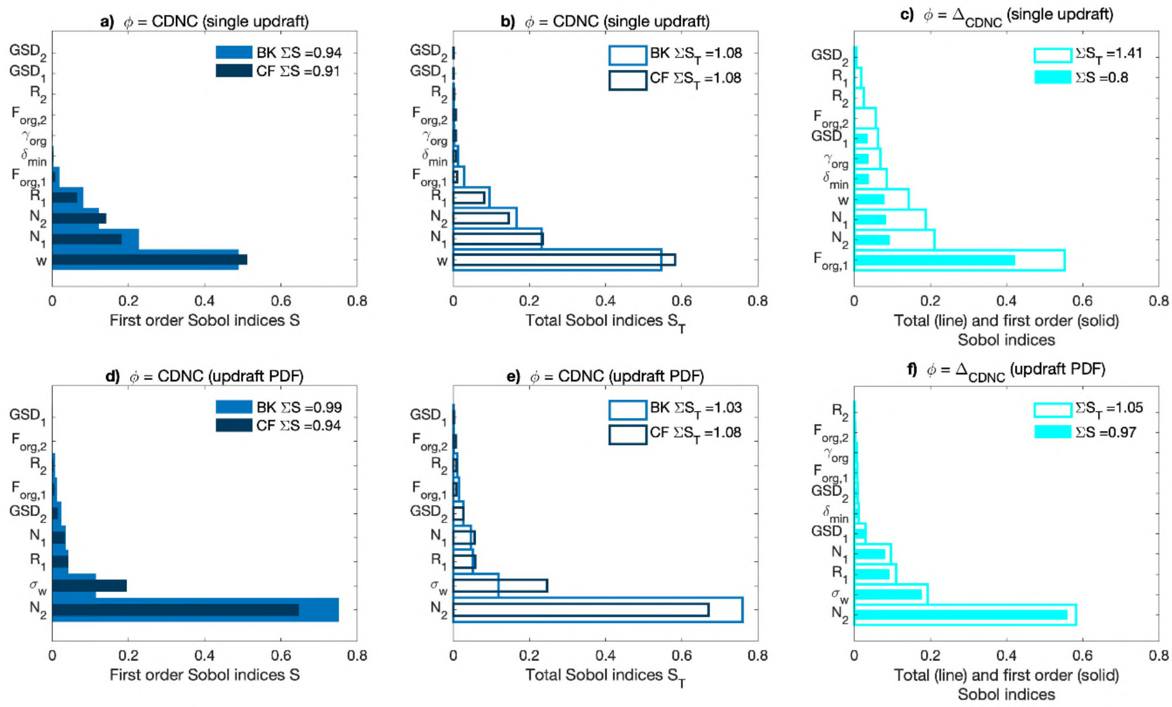

Supplementary Figure 8. Sobol parameter sensitivity rankings for marine (MA) setup using parameter uncertainty ranges in Supplementary Table 1. a) First order Sobol indices S evaluated on model variance in cloud droplet number concentration (CDNC) at 30 m above maximum supersaturation using bulk Köhler (blue, $SR_{BK,w}$) and approximate compressed film models (dark blue, $SR_{CF,w}$). The cloud parcel model initialised with fixed updraft *w*. **b)** As in a) but for total Sobol indices S_T. **c)** $SR_{\Delta,w}$ Total (line) and first order (solid) Sobol indices evaluated on model variance in relative percentage change in CDNC at 30 m above maximum supersaturation, $\Delta_{\mathrm{CDNC}} = (\mathrm{CDNC_{CF}}/\mathrm{CDNC_{BK}} - 1) \times 100\%$. **d-f)** as in a-c) but with an updraft probability density function of width σ_w used in prediction of average CDNC (Eqs. S1 and S2); $SR_{BK,\sigma}$, $SR_{CF,\sigma}$ and $SR_{\Delta,\sigma}$.

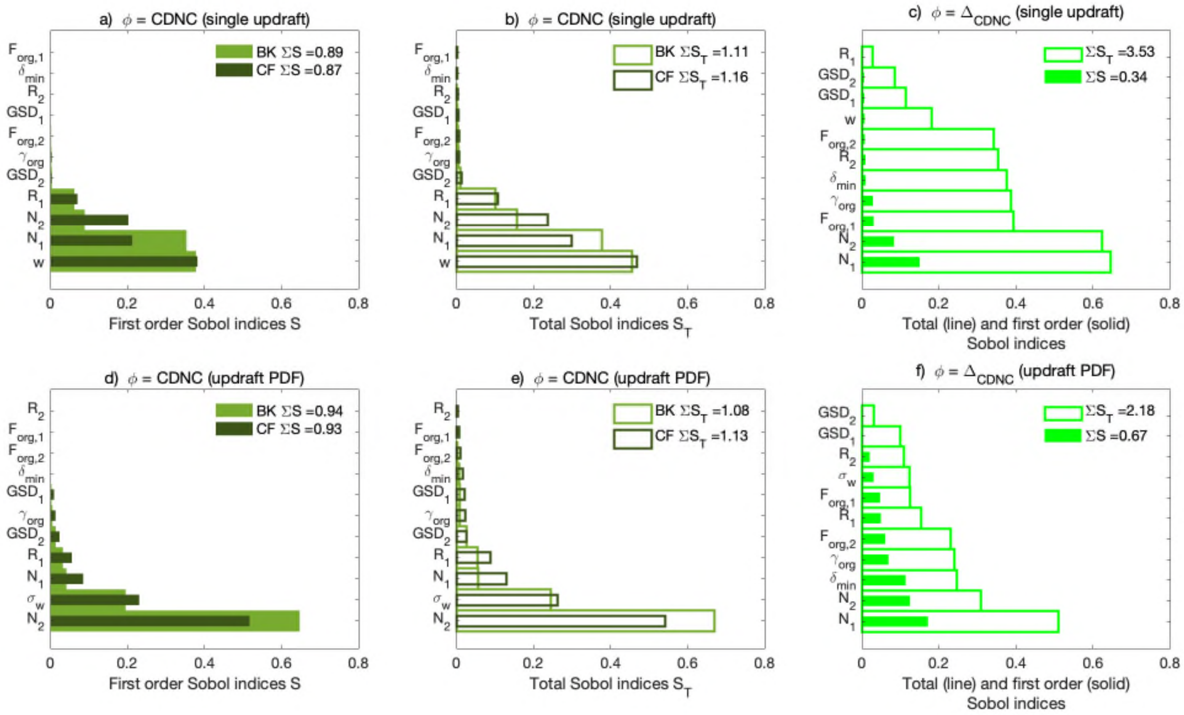

Supplementary Figure 9. Sobol parameter sensitivity rankings for boreal (HYY) setup using parameter uncertainty ranges in Supplementary Table 1. a) First order Sobol indices S evaluated on model variance in cloud droplet number concentration (CDNC) at 30 m above maximum supersaturation using bulk Köhler (blue, $SR_{BK,w}$) and approximate compressed film models (dark blue, $SR_{CF,w}$). The cloud parcel model initialised with fixed updraft *w*. **b)** As in a) but for total Sobol indices S_T. **c)** $SR_{\Delta,w}$ Total (line) and first order (solid) Sobol indices evaluated on model variance in relative percentage change in CDNC at 30 m above maximum supersaturation, $\Delta_{CDNC} = (CDNC_{CF}/CDNC_{BK} - 1) \times 100\%$. **d-f)** as in a-c) but with an updraft probability density function of width σ_w used in prediction of average CDNC (Eqs. S1 and S2); $SR_{BK,\sigma}$, $SR_{CF,\sigma}$ and $SR_{\Delta,\sigma}$.

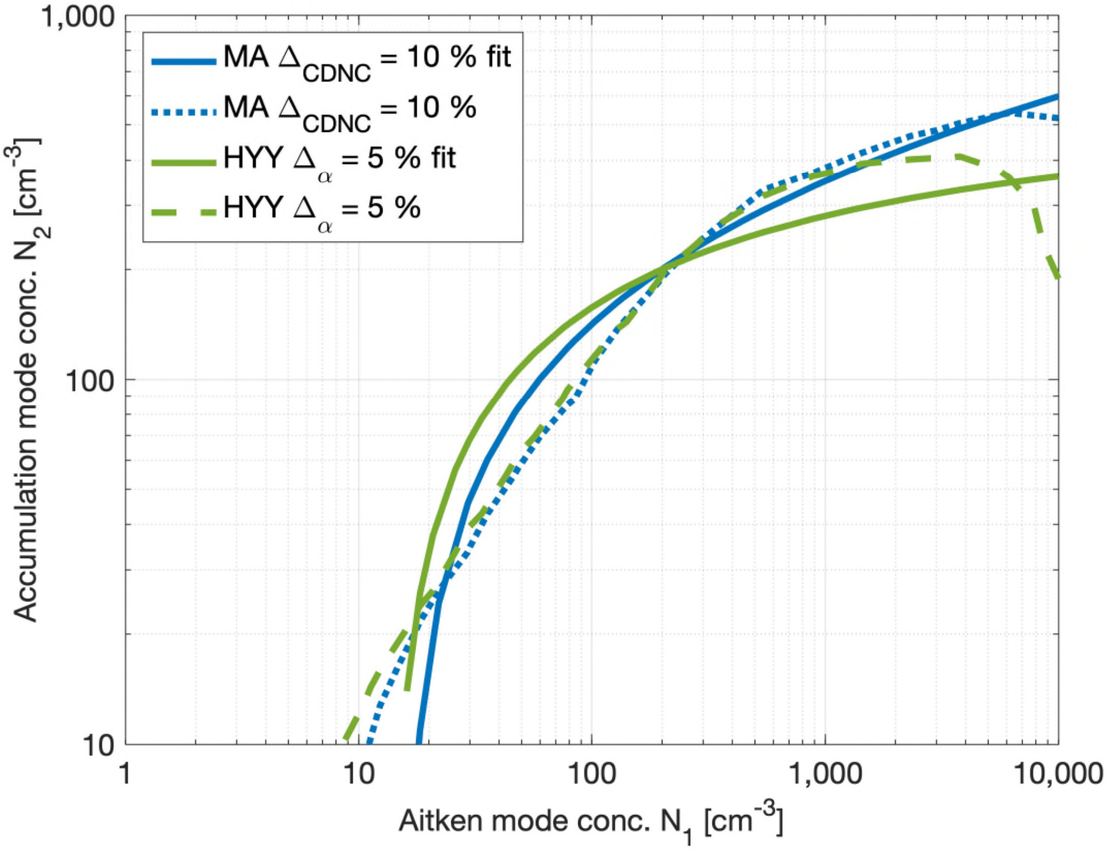

Supplementary Figure 10. Fitted power law criteria on Aitken and accumulation mode concentrations for significant cloud microphysical responses to the surface phase. Functional forms are tabulated in the main text.

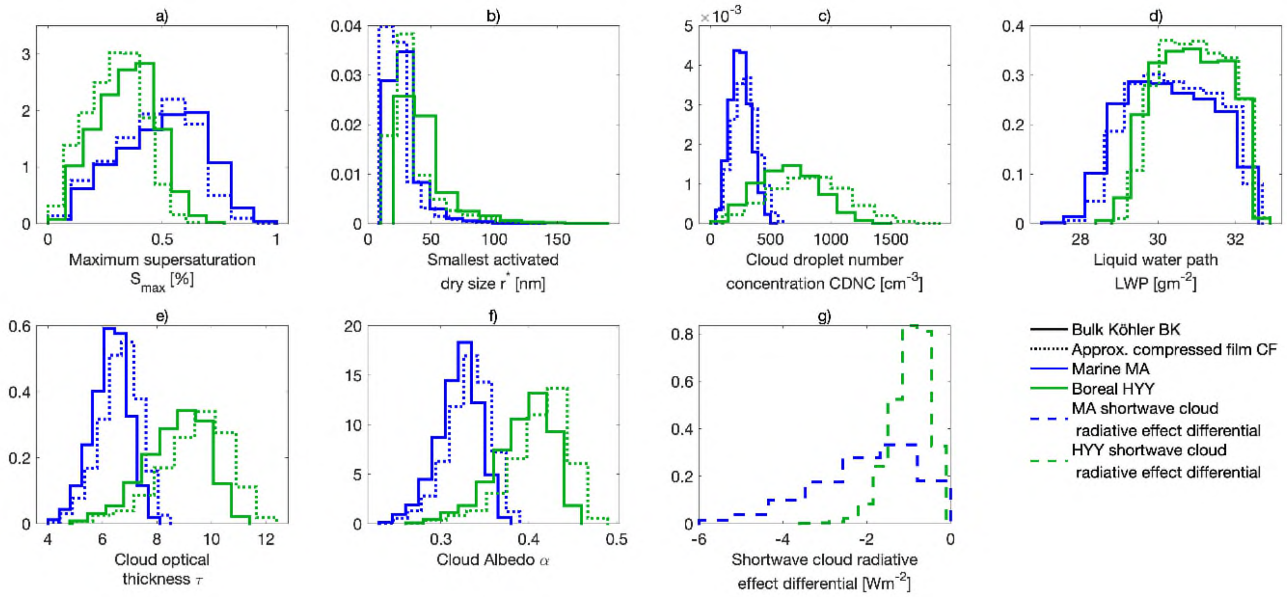

Supplementary Figure 11. Histogram probability density function distributions of parcel model predicted microphysical and optical properties (a-g, the properties given on the x-axes) from 5,000 Latin hypercube sampled input parameter combinations from ranges given in Table 2. Simulations are performed assuming a cloud depth of 200 m and fixed average cloud fractions of 0.7 and 0.5 for marine and boreal cases, respectively.

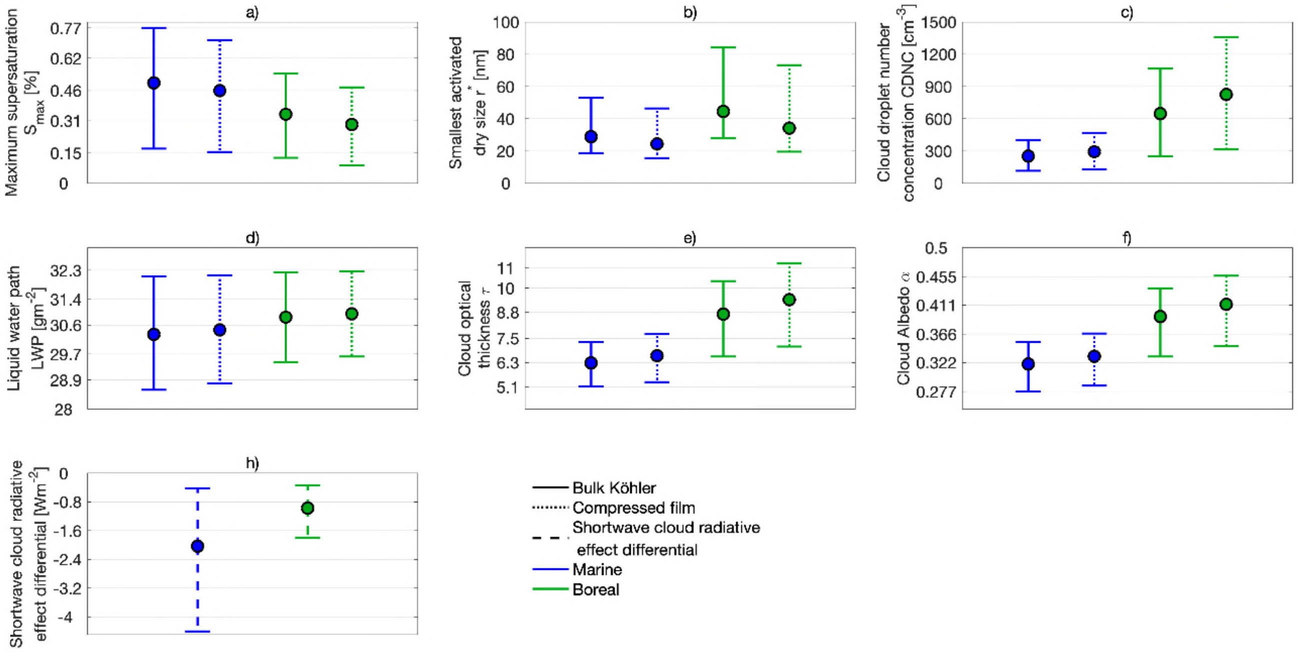

Supplementary Figure 12. Parametric uncertainties in parcel model predictions of cloud microphysical and optical properties (a-h, properties given on the y-axes) determined from 95th-5th percentiles of outputs produced from globally Latin hypercube sampled parameter combinations in ranges listed in Table 2. Simulations are performed assuming a cloud depth of 200 m and fixed average cloud fractions of 0.7 and 0.5 for marine and boreal cases, respectively.

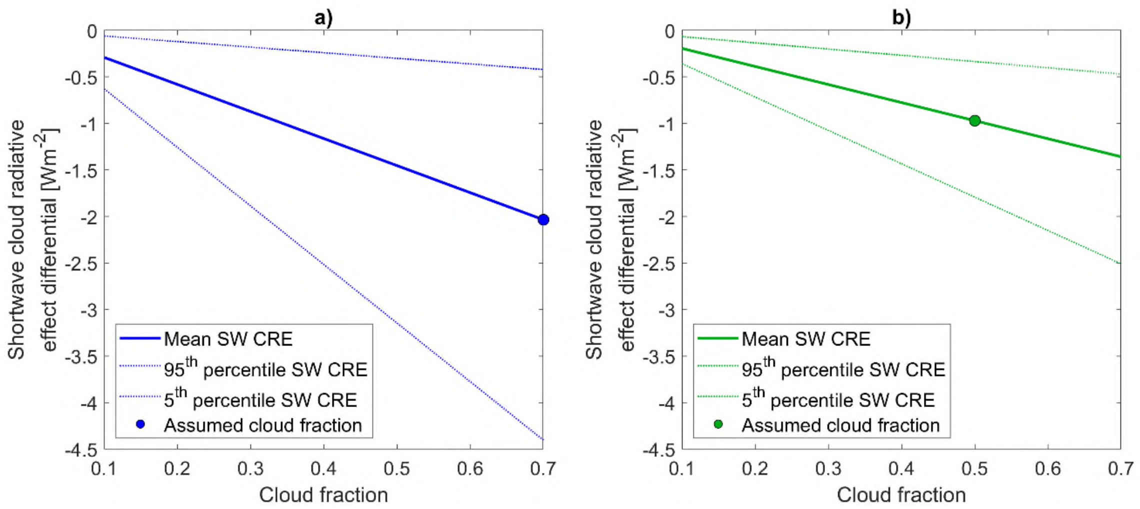

Supplementary Figure 13. Dependence of the shortwave cloud radiative effect (SW-CRE) differential and its parametric uncertainty on the fixed average cloud fraction used in Supplementary Equation 3 for a) marine and b) boreal, land-proxy setups (Table 2). Dots indicate the mean SW-CRE differential obtained from distributions given in Supplementary Fig. 11 using the respective fixed cloud fractions.

Location	Mace Head		Hyytiälä	
Characteristic environment	Marine		Boreal	
Parameter	Min	Max	Min	Max
Aitken mode number concentration N_1 [cm^{-3}]	150	600	440	1780
Acc. mode number concentration N_2 [cm^{-3}]	60	250	160	920
Aitken mode geometric mean radius R_1 [nm]	15.5	23.5	14	31
Acc. mode geometric mean radius R_2 [nm]	70.0	100.0	66	98.5
Geom. standard deviation of Aitken mode $\sigma_{g,1}$	1.40	1.60	1.56	1.95
Geom. standard deviation of acc. mode $\sigma_{g,2}$	1.40	1.60	1.46	1.78
Organic fraction in Aitken mode $F_{org,1}$	0.05	0.40	0.45	0.75
Organic fraction in acc. mode $F_{org,2}$	0.05	0.40	0.45	0.75
Updraft velocity w [$m\ s^{-1}$]	0.05	2.40	0.05	2.40
Updraft PDF standard deviation σ_w [$m\ s^{-1}$]	0.2	0.6	0.2	0.6
Compressed film minimum thickness δ_{min} [nm]	0.16	0.30	0.16	0.30
Organic component surface tension γ_{org} [$mN\ m^{-1}$]	30	50	30	50

Supplementary Table 1: Table of parameter ranges probed with the Sobol algorithm (Supplementary Figs. 8 and 9).

Supplementary references

1: McCormick, R. and Ludwig, J. H. Climate Modification by Atmospheric Aerosols. *Science*, **156**, 1358–1359, doi:10.1126/science.156.3780.1358, (1967).

2. Twomey, S. Pollution and the planetary albedo. *Atmos. Environ.*, **8**, 1251–1256, (1974).

3.Kanakidou, M. *et al.* Organic aerosol and global climate modelling: a review. *Atmos. Chem. Phys.*, **5**, 1053-1123, https://doi.org/10.5194/acp-5-1053-2005, (2005).

4. IPCC, 2013: Climate Change 2013: The Physical Science Basis. Contribution of Working Group I to the Fifth Assessment Report of the Intergovernmental Panel on Climate Change [Stocker, T.F., D. Qin, G.-K. Plattner, M. Tignor, S.K. Allen, J. Boschung, A. Nauels, Y. Xia, V. Bex and P.M. Midgley (eds.)]. Cambridge University Press, Cambridge, United Kingdom and New York, NY, USA, 1535 pp.

5. Tsigaridis, K. *et al.* The AeroCom evaluation and intercomparison of organic aerosol in global models. *Atmos. Chem. Phys.*, **14**, 10845-10895, https://doi.org/10.5194/acp-14-10845-2014, (2014).

6. Köhler, H. The nucleus in and the growth of hygroscopic droplets. *T. Faraday Soc.*, **32**, 1152–1161, doi:10.1039/TF9363201152, (1936).

7. Shantz, N. C., Leaitch, W. R., and Caffrey, P. F. Effect of organics of low solubility on the growth rate of cloud droplets. *J. Geophys. Res.*, **108**, 4168, doi:10.1029/2002JD002540, (2003).

8. Quinn, P. K., Bates, T. S., Coffman, D. J., and Covert, D. S. Influence of particle size and chemistry on the cloud nucleating properties of aerosols. *Atmos. Chem. Phys.*, **8**, 1029-1042, https://doi.org/10.5194/acp-8-1029-2008, (2008).

9. Shulman, M. L., Jacobson, M. C., Charlson, R. J., Synovec, R. E, and Young, T. E. Dissolution behaviour and surface tension effects of organic compounds in nucleating cloud droplets. *Geophys. Res. Lett.*, **23**, 277–280, (1996).

10. Prisle, N. L., Raatikainen, T., Laaksonen, A., and Bilde, M. Surfactants in cloud droplet activation: mixed organic-inorganic particles. *Atmos. Chem. Phys.*, **10**, 5663–5683, doi:10.5194/acp-10- 5663-2010, (2010).

11. Werner, J. *et al.* Surface partitioning in organic-inorganic mixtures contributes to the size-dependence of the phase-state of atmospheric nanoparticles. *Environ. Sci. Technol.*, **50** (14), 7434–7442, doi:10.1021/acs.est.6b00789 (2016).

12. Lowe, S., Partridge, D. G., Topping, D., and Stier, P. Inverse modelling of Köhler theory – Part 1: A response surface analysis of CCN spectra with respect to surface-active organic species. *Atmos. Chem. Phys.*, **16**, 10941-10963, https://doi.org/10.5194/acp-16-10941-2016, (2016).

13. Ruehl, C. R. *et al.* Strong evidence of surface tension reduction in microscopic aqueous droplets. *Geophys. Res. Lett.* **39**, L23801, (2012).

14. Ruehl, C. R., Davies, J. F., and Wilson, K. R. An interfacial mechanism for cloud droplet formation on organic aerosols. *Science*, **351**, 1447–1450, doi:10.1126/science.aad4889, (2016).

15. Nguyen, Q. T., Kjær, K. H., Kling, K. I., Boesen, T., and Bilde, M. Impact of fatty acid coating on the CCN activity of sea salt particles. *Tellus B: Chemical and Physical Meteorology,* **69:**1, DOI: 10.1080/16000889.2017.1304064 (2017).

16. Ovadnevaite, J. *et al.* Surface tension prevails over solute effect in organic-influenced cloud droplet activation. *Nature* **546**, 637–641, (2017).

17. Sorjamaa, R. *et al.* The role of surfactants in Köhler theory reconsidered. *Atmos. Chem. Phys*., **4**, 2107–2117, doi:10.5194/acp-4-2107-2004, (2004).

18. Topping, D. An analytical solution to calculate bulk mole fractions for any number of components in aerosol droplets after considering partitioning to a surface layer. *Geosci. Model Dev.*, **3**, 635– 642, doi:10.5194/gad-3-635-2010, (2010).

19. Prisle, N. L. *et al.* Surfactant effects in global simulations of cloud droplet activation. *Geophys. Res. Lett.*, **39**, L05802, doi:10.1029/2011GL050467 (2012).

20. Nenes, A., R. J. *et al.* Can chemical effects on cloud droplet number rival the first indirect effect? *Geophys. Res. Lett.*, **29**(17), 1848, doi:10.1029/2002GL015295, (2002).

21. Fountoukis, C. and Nenes, A. Continued development of a cloud droplet formation

parameterization for global climate models. *J. Geophys. Res.*, **110**, D11212, doi:10.1029/2004JD005591 (2005).

22. Ghan, S. J. *et al.* Droplet Nucleation: Physically-Based Parameterization and Validation. *Journal of Advances in Modeling Earth Systems*, **3**, 1-33 (2011).

23. Jimenez, J. L. et al. Evolution of organic aerosols in the atmosphere. *Science*, **326**, 1525–1529 (2009).

24. Asmi, E. *et al.* Secondary new particle formation in Northern Finland Pallas site between the years 2000 and 2010. *Atmos. Chem. Phys.*, **11**, 12959–12972, doi:10.5194/acp-11-12959-2011, (2011).

25. Sobol, I. M. Sensitivity analysis for non-linear mathematical models. *Mathematical Modelling and Computational Experiment 1* 407–414 (1993); Translated from Russian: I.M. Sobol', Sensitivity estimates for nonlinear mathematical models, *Matematicheskoe Modelirovanie 2* 112–118 (1990).

26. Sobol, I. M. Global sensitivity indices for nonlinear mathematical models and their Monte Carlo estimates. *Math. Comput. Simul.*, **55**, 271–280, doi:10.1016/S0378-4754(00)00270-6, (2001).

27. Saltelli, A. *et al.* Variance based sensitivity analysis of model output. Design and estimator for the total sensitivity index. *Comput. Phys. Commun.*, **181**, 259–270, doi:10.1016/j.cpc.2009.09.018, (2010).

28. Loeb, N. G. *et al.* Clouds and the Earth's Radiant Energy System (CERES) Energy Balanced and Filled (EBAF) top-of-atmosphere (TOA) Edition-4.0 data product. *J. Climate*, **31**, 895–918, https://doi.org/10.1175/JCLI-D-17-0208.1, (2018).

29. Topping, D., Connolly, P., McFiggans, G. Cloud Droplet Number Enhanced by Co-Condensation of Organic Vapours. *Nat. Geosci*. **6**, 443– 446, (2013).

30. O'Dowd, C. D. *et al.* Biogenically driven organic contribution to marine aerosol. *Nature*, **431**, 676–680, (2004).

31. Tunved, P. *et al.* Aerosol characteristics of air masses in northern Europe: Influences of location, transport, sinks, and sources. *J. Geophys. Res.*, **110**, D07201, doi:10.1029/2004JD005085, (2005).

32. Tørseth, K., *et al.* Introduction to the European Monitoring and Evaluation Programme (EMEP) and observed atmospheric composition change during 1972–2009, *Atmos. Chem. Phys.*, **12**, 5447-5481, https://doi.org/10.5194/acp-12-5447-2012, (2012).

33. O'Dowd, C., *et al.* Do anthropogenic, continental or coastal aerosol sources impact on a marine aerosol signature at Mace Head? *Atmos. Chem. Phys.*, **14**, 10687-10704, https://doi.org/10.5194/acp-14-10687-2014, (2014).

34. Partridge, D. G. *et al.* Inverse modelling of cloud-aerosol interactions – Part 2: Sensitivity tests on liquid phase clouds using a Markov chain Monte Carlo based simulation approach. *Atmos. Chem. Phys.*, **12**, 2823-2847, https://doi.org/10.5194/acp-12-2823-2012, (2012).

35. Bender, F. A.-M., Rodhe, H., Charlson, R. J., Ekman, A. M.-L. and Loeb, N. 22 views of the global albedo—comparison between 20 GCMs and two satellites. *Tellus A*, **58** 320–330, (2006).

36. Sipilä, M. *et al.* Molecular-scale evidence of aerosol particle formation via sequential addition of HIO3. *Nature*, **537**, 532–534, https://doi.org/10.1038/nature19314, (2016).

37. Freud, E. *et al*. Pan-Arctic aerosol number size distributions: seasonality and transport patterns, *Atmos. Chem. Phys.*, **17**, 8101-8128, https://doi.org/10.5194/acp-17-8101-2017, (2017).

38. McKay, M. D., Beckman R. J., and Conover, W. J. A comparison of three methods for selecting values of input variables in the analysis of output from a computer code. *Technometrics*, **21**, 239–245 (1979).

39. Rastak, N., *et al.* Microphysical explanation of the RH-dependent water affinity of biogenic organic aerosol and its importance for climate, *Geophys. Res. Lett.*, **44**, 5167–5177, https://doi.org/10.1002/2017gl073056, (2017).

40. McCoy, D. T. *et al.* Natural aerosols explain seasonal and spatial patterns of Southern Ocean cloud albedo. *Sci. Adv.*, **1**, e1500157, doi: https://doi.org/10.1126/sciadv.1500157 (2015).

41. Murphy, B. N., Julin, J., Riipinen, I., and Ekman, A. M. L. Organic aerosol processing in

tropical deep convective clouds: Development of a new model (CRM-ORG) and implications for sources of particle number. *J. Geophys. Res.*, **120**, 10441–10464, https://doi.org/10.1002/2015JD023551, (2015).

42. Andreae, M. O. *et al.* Aerosol characteristics and particle production in the upper troposphere over the Amazon Basin. , *Atmos. Chem. Phys.*, **18**, 921-961, https://doi.org/10.5194/acp-18-921-2018, (2018).

43. Forestieri, S. D. *et al.* Establishing the impact of model surfactants on cloud condensation nuclei activity of sea spray aerosol mimics, *Atmos. Chem. Phys.*, **18**, 10985–11005, https://doi.org/10.5194/acp-18-10985-2018, (2018).

44. Dusek, U. *et al.* Size matters more than chemistry for cloud-nucleating ability of aerosol particles. *Science*, **312**(5778), 1375–1378, (2006).

45. Roelofs, G. J. and Jongen, S. A model study of the influence of aerosol size and chemical properties on precipitation formation in warm clouds. *J. Geophys. Res.*, **109**, D22201, doi:10.1029/2004JD004779, (2004).

46. Partridge, D. G. *et al.* Inverse modeling of cloud-aerosol interactions – Part 1: Detailed response surface analysis, *Atmos. Chem. Phys.*, **11**, 7269-7287, https://doi.org/10.5194/acp-11-7269-2011, (2011).

47. Lowe, P. R. An approximating polynomial for the computation of saturation vapor pressure. *J. Appl. Meteor.*, **16**, 100–103, (1977).

48. Hanel, G. The role of aerosol properties during the condensational stage of cloud: a reinvestigation of numerics and microphysics. *Beitr. Phys. Atmosph.*, **60**, 321–339, (1987).

49. Pruppacher, H. R. and Klett, J. D. Microphysics of Clouds and Precipitation. *Springer*, (1997).

50. Fukuta, N. & Walter, A. Kinetics of hydrometeor growth from a vapor-spherical model. *J. Atmos. Sci.* **27**, 1160–1172 (1970).

51. Seinfeld, J. H., Pandis, S. N. Atmospheric chemistry and physics: from air pollution to climate change, 2nd edition. *Wiley*, (2006).

52. Yoon, Y. J., *et al.* Seasonal characteristics of the physicochemical properties of North Atlantic marine atmospheric aerosols. *J. Geophys. Res.*, **112**, D04206, doi:10.1029/2005JD007044, (2007).

53. Ovadnevaite, J., *et al.* Submicron NE Atlantic marine aerosol chemical composition and abundance: Seasonal trends and air mass categorization, *J. Geophys. Res. Atmos.*, **119**, 11850–11863, doi:10.1002/2013JD021330, (2014).

54. Zieger, P. *et al.* Revising the hygroscopicity of inorganic sea salt particles, *Nat. Commun.*, **8**, 15883, https://doi.org/10.1038/ncomms15883, (2017).

55. Tervahattu, H. *et al.* New evidence of an organic layer on marine aerosols:, *J. Geophys.*

Res. 107(D7), **4053**, doi:10.1029/2000JD000282, (2002).

56. Äijälä, M., *et al.* Constructing a data-driven receptor model for organic and inorganic aerosol – a synthesis analysis of eight mass spectrometric data sets from a boreal forest site, *Atmos. Chem. Phys.*, **19**, 3645-3672, https://doi.org/10.5194/acp-19-3645-2019, (2019).

57. Ehn, M., *et al.* A large source of low-volatility secondary organic aerosol, *Nature*, **506**, 476–479, (2014).

58. Heintzenberg, J., Covert, D., and Van Dingenen, R.: Size distribution and chemical composition of marine aerosols: a compilation and review, *Tellus B*, **52**, 1104–1122, doi:10.1034/j.1600- 0889.2000.00136.x, (2000).

59. Stephens, G. L. Radiation profiles in extended water clouds. II: parameterization schemes. *J. Atmos. Sci.* **35**, 2123–2132, (1978).

60. Albrecht, B. A., Fairall, C., Thomson, D., White, A., and Snider, J. Surface-based remote sensing of the observed and the adiabatic liquid water content. *Geophys. Res. Lett.*, **17**, 89–92, doi:10.1029/GL017i001p00089, (1990).

61. Hansen, J. and Travis, L. D. Light scattering in planetary atmospheres. *Space Sci. Rev.*, **16**, 527–610, (1974).

62. Bohren, C. F. Multiple scattering of light and some of its observable consequences. *Am. J. Phys.* **55**, 524–533, doi:10.1119/1.15109, (1987).

63. Kravitz, B., Wang, H., Rasch, P. J., Morrison, H., Solomonm, A. B. Process-model simulations of cloud albedo enhancement by aerosols in the Arctic. *Phil. Trans. R. Soc. A* **372**, 20140052, doi:10.1098/rsta.2014.0052, (2014).

64. Schwartz, S. E. & Slingo, A. Clouds, Chemistry, and Climate (eds Crutzen, P. & Ramanathan, V.). *Springer*, 191–236, (1996).

65. Latham, J. *et al.* Global temperature stabilization via controlled albedo enhancement of low-level maritime clouds. *Phil. Trans. R. Soc. A,* 366, 3969–3987 (2008).

66. Eastman, R., Warren, S. G., and Hahn, C. J. Variations in Cloud Cover and Cloud Types over the Ocean from Surface Observations, 1954–2008. *J. Clim.*, **24**, 5914–5934, doi:10.1175/2011JCLI3972.1, (2011).

67. Hussein, et al. Evaluation of an automatic algorithm for fitting the particle number size distributions. *Boreal Env. Res.*, **10**: 337–355, (2005).

68. West, R. E. L. *et al.* The importance of vertical velocity variability for estimates of the indirect aerosol effects. *Atmos. Chem. Phys.*, **14**, 6369-6393, https://doi.org/10.5194/acp-14-6369-2014, (2014).

69. Homma, T. and A. Saltelli. Importance measures in global sensitivity analysis of nonlinear models. *Reliability Engineering and System Safety*, **52**, 1–17, (1996).

70. Morales, R., and Nenes, A. Characteristic updrafts for computing distribution-averaged cloud droplet number and stratocumulus cloud properties. *J. Geophys. Res.*, **115**, D18220, doi:10.1029/2009JD013233, (2010).

71. Meskhidze, N., A. Nenes, W. C. Conant, and J. H. Seinfeld, Evaluation of a new cloud droplet activation parameterization with in situ data from CRYSTAL-FACE and CSTRIPE. *J. Geophys. Res.*, **110**, D16202, doi:10.1029/2004JD005703, (2005).

72. Fountoukis, C., *et al.* Aerosol-cloud drop concentration closure for clouds sampled during the International Consortium for Atmospheric Research on Transport and Transformation 2004 campaign. *J. Geophys. Res.*, **112**, D10S30, doi:10.1029/2006JD007272, (2007).
